# Hospital Secretaries and Their Salaries

**Published:** 1896-01-11

**Authors:** 


### HOSPITAL SECRETARIES AND THEIR SALARIES.

### A PROTEST.

H. H. G., the Secretary of a London hospital, writes: "From a report in the *Daily Chronicle* of December 31st it appears that at a meeting of governors of the Great Northern Central Hospital, Mr. Murdoch, M.P., stated from the chair that the average amount paid to the late secretary, Mr. Grant, 'was exceeded by that paid by other London hospitals to their secretaries,' and the statement is reported to have been received with cries (or a cry) of 'Shame!' In an earlier part of the same report the average amount paid to Mr. Grant is stated to be 'equal to £750 per annum.' Mr. Murdoch's statement, as it appears, is one which I think might well be publicly contradicted, for, apart from any question as to the size of the hospital, I should very much doubt if there are six hospital secretaries in London who receive as much as £700 a year. It is a question as to which the Hospitals Association has, possibly, accurate information, and which, I venture to think, one of its chief officials might very well challenge, or, at least, might write to Mr. Murdoch asking him for his authority, if the report is a correct one. As it stands, the report is of course, likely to inflict great injury on hospitals generally, which, I am sure, would be far from Mr. Murdoch's wishes."

*** We call special attention to the above letter which we publish, from a hospital secretary, on the subject of certain remarks made by Mr. Murdoch at the meeting of the governors of the Great Northern Central Hospital. We find that there is a good deal of feeling abroad on the subject of these remarks, so far as they relate to the salaries paid to hospital secretaries, and we feel that Mr. Murdoch should either give chapter and verse for the declaration that a salary of £750 is less than the average amount paid to hospital secretaries or that the statement should be unreservedly withdrawn. We have often expressed our view that hospital secretaries are under, and not over paid, believing as we do, that within well-defined limits, the higher the salary the more prosperous will be the particular institution which has the wisdom to select a good man and pay him adequately.—ED. *T. H.*

